# Severe hyperkalemia requiring hospitalization: predictors of mortality

**DOI:** 10.1186/cc11872

**Published:** 2012-11-21

**Authors:** Jung Nam An, Jung Pyo Lee, Hee Jung Jeon, Do Hyoung Kim, Yun Kyu Oh, Yon Su Kim, Chun Soo Lim

**Affiliations:** 1 Department of Internal Medicine, Seoul National University College of Medicine, 101 Daehang-ro, Jongro-gu, Seoul 110-744, Republic of Korea; 2 Department of Internal Medicine, Seoul National University Boramae Medical Center, 20 Boramae-ro 5-gil, Dongjak-gu, Seoul 156-707, Republic of Korea

## Abstract

**Introduction:**

Severe hyperkalemia, with potassium (K^+^) levels ≥ 6.5 mEq/L, is a potentially life-threatening electrolyte imbalance. For prompt and effective treatment, it is important to know its risk factors, clinical manifestations, and predictors of mortality.

**Methods:**

An observational cohort study was performed at 2 medical centers. A total of 923 consecutive Korean patients were analyzed. All were 19 years of age or older and were hospitalized with severe hyperkalemia between August 2007 and July 2010; the diagnosis of severe hyperkalemia was made either at the time of admission to the hospital or during the period of hospitalization. Demographic and baseline clinical characteristics at the time of hyperkalemia diagnosis were assessed, and clinical outcomes such as in-hospital mortality were reviewed, using the institutions' electronic medical record systems.

**Results:**

Chronic kidney disease (CKD) was the most common underlying medical condition, and the most common precipitating factor of hyperkalemia was metabolic acidosis. Emergent admission was indicated in 68.6% of patients, 36.7% had electrocardiogram findings typical of hyperkalemia, 24.5% had multi-organ failure (MOF) at the time of hyperkalemia diagnosis, and 20.3% were diagnosed with severe hyperkalemia at the time of cardiac arrest. The in-hospital mortality rate was 30.7%; the rate was strongly correlated with the difference between serum K^+ ^levels at admission and at their highest point, and with severe medical conditions such as malignancy, infection, and bleeding. Furthermore, a higher in-hospital mortality rate was significantly associated with the presence of cardiac arrest and/or MOF at the time of diagnosis, emergent admission, and intensive care unit treatment during hospitalization. More importantly, acute kidney injury (AKI) in patients with normal baseline renal function was a strong predictor of mortality, compared with AKI superimposed on CKD.

**Conclusions:**

Severe hyperkalemia occurs in various medical conditions; the precipitating factors are similarly diverse. The mortality rate is especially high in patients with severe underlying disease, coexisting medical conditions, and those with normal baseline renal function.

## Introduction

Potassium (K^+^) is a ubiquitous cation contained mostly within the intracellular fluid; only about 2% of total body K^+ ^is found in the extracellular fluid [1]. In healthy humans, serum K^+ ^levels are tightly controlled within the narrow range of 3.5 to 5.0 mEq/L [2], thus retaining a normal ratio between the intracellular and extracellular compartments. This homeostasis plays a critical role in maintaining cellular resting membrane potential and neuromuscular function and is essential for normal activity of muscles, nerves, and the heart [3]. Hyperkalemia, resulting from an imbalance in K^+ ^homeostasis, is defined as a serum K^+ ^level of greater than 5.0 mEq/L and is further classified as mild, moderate, or severe [4,5]. It has been reported that drug therapy and impaired renal function are the main factors predisposing to the development of hyperkalemia [6-8].

Severe hyperkalemia (K^+ ^of at least 6.5 mEq/L) is a potentially life-threatening electrolyte disorder [9] that has been reported to occur in 1% to 10% of all hospitalized patients, a higher percentage than that seen in outpatients [10,11]. It is associated with electrocardiogram (ECG) abnormalities, including peaked T waves, shortened QT intervals, prolonged PR intervals, reduction in the amplitude of P waves, and 'sine-wave' ventricular rhythms with wide QRS complexes. Severe hyperkalemia eventually causes fatal arrhythmias such as ventricular fibrillation or asystole, leading to cardiac arrest [12-15]. Severe hyperkalemia is a medical emergency and can lead to significant morbidity and mortality; it therefore requires hospitalization, ECG monitoring, and immediate treatment [16].

To promptly and effectively treat severe hyperkalemia, it is important to know the risk factors, the clinical manifestations, the therapeutic approaches, and the factors that predict both mortality and improvement in this disorder [17-19]. Although most of these factors are well documented, reliable predictors of clinical outcomes such as in-hospital mortality have not been established. We therefore designed this study to identify common factors predisposing to severe hyperkalemia and to analyze the relationship between serum K^+ ^levels and clinical outcomes, including in-hospital mortality. Furthermore, we attempted to determine the association between in-hospital mortality and multiple clinical factors in patients with severe hyperkalemia.

## Materials and methods

### Study population

This observational cohort study was performed in two medical centers during a 3-year period. The institutions involved were Seoul National University Hospital (Seoul, Korea) and Seoul National University Boramae Medical Center (Seoul, Korea), which are tertiary referral hospitals with 1,600 and 800 beds, respectively, and an academic affiliation with Seoul National University College of Medicine. Using the electronic medical record system, we identified the population of hospitalized patients at these centers between August 2007 and July 2010; we enrolled patients at or over the age of 19 years who had at least one severe hyperkalemic event, with serum K^+ ^levels of at least 6.5 mEq/L. In patients who had several of these events, the first event was used for analysis. All cases of severe hyperkalemia were diagnosed either at the time of admission to the hospital or during the period of hospitalization. This study was approved by the institutional review boards of both hospitals; the need for informed consent was waived because of the study's retrospective design. All clinical investigations were conducted in accordance with the guidelines of the 2008 Declaration of Helsinki.

### Data collection

Detailed evaluations of hospitalizations, prescriptions, and laboratory findings were performed for all identified patients by using the electronic medical record systems of the institutions. Data, including patients' medical histories, comorbid diseases, medications, coexisting medical conditions, ECG findings, and hyperkalemia management strategies, were abstracted from admission records, progress records, nursing records, discharge summaries, and the records of the emergency department and the intensive care unit (ICU). The type of admission and the timing of the onset of hyperkalemia were reviewed; in patients with hospital-acquired hyperkalemia, the period from admission to diagnosis and the hospital location at diagnosis were also reviewed. The symptoms associated with hyperkalemia and the occurrence of multi-organ failure (MOF) or cardiac arrest (or both) at the time of severe hyperkalemia diagnosis were examined closely.

Chronic kidney disease (CKD) was classified into five groups on the basis of the estimated glomerular filtration rate (eGFR): normal renal function and stage I CKD, eGFR of at least 90 mL/minute per 1.73 m^2^; stage II, eGFR of 60 to 89 mL/minute per 1.73 m^2^; stage III, eGFR of 30 to 59 mL/minute per 1.73 m^2^; stage IV, eGFR of 15 to 29 mL/minute per 1.73 m^2^; and stage V, eGFR of less than 15 mL/minute per 1.73 m^2 ^or requiring renal replacement therapy (RRT). Patients with a previous diagnosis of severe hyperkalemia were considered to have recurrent severe hyperkalemia. Hypertension was defined as a systolic blood pressure of greater than 140 mm Hg or a diastolic pressure of greater than 90 mm Hg or by the use of antihypertensive drugs. Diabetes mellitus was diagnosed in patients with a random blood glucose concentration of greater than 200 mg/dL, a fasting plasma glucose level of greater than 126 mg/dL on at least two separate occasions, or a glycated hemoglobin of greater than 7.0% or by the use of oral hypoglycemic agents or insulin. Cirrhosis was defined by computed tomography or sonography, and congestive heart failure was defined as a New York Heart Association functional class III or IV. Coronary artery disease was defined by a prior diagnosis of ischemic heart disease and positive ECG findings; pulmonary diseases included tuberculosis, chronic obstructive pulmonary disease, and asthma.

We identified prescriptions for the most common medications that are capable of increasing serum K^+ ^levels: angiotensin-converting enzyme inhibitors, angiotensin II receptor blockers, potassium-sparing diuretics, beta blockers, non-steroidal anti-inflammatory drugs, digoxin, and potassium supplements. These medications were considered potentially contributory if administered within 24 to 36 hours of the onset of hyperkalemia; if patients were already on dialysis, these medications were considered noncontributory.

Coexisting medical conditions affecting the occurrence of severe hyperkalemia were categorized into one of three groups: those causing renal impairment, those causing K^+ ^shift across cell membranes, and others. The initial categorization was performed by electronic medical record review and confirmed on the basis of the clinical judgment of the researchers. Acute kidney injury (AKI) was defined by the Acute Kidney Injury Network criteria and consisted of an absolute increase in serum creatinine of at least 0.3 mg/dL, a percentage increase in the serum creatinine of at least 50%, and/or a reduction in urine output, defined as an output of less than 0.5 mL/kg per hour for greater than 6 hours; these changes were required to occur over a rapid time course (< 48 hours) to meet the definition of AKI [20,21]. The diagnosis of infection required not only at least two signs of systemic inflammatory response syndrome but also clinical evidence of infection. Volume depletion was defined as a clinical situation resulting from decreased effective circulating volume and total extracellular fluid volume or as decreased effective circulating volume with increased total extracellular fluid volume. Bleeding was defined as class II or higher hemorrhage on the basis of Advanced Trauma Life Support guidelines, or grade 2 or higher hemorrhage on the basis of World Health Organization guidelines, with definite clinical signs and symptoms. Metabolic acidosis was defined as an arterial pH of less than 7.35. Poor compliance with K^+^-lowering agents was determined by review of the attending physician's records, including the patient's medical history, and the reviewer's judgment.

When available, ECGs corresponding to the time of severe hyperkalemia diagnosis or those nearest in time to the diagnosis were reviewed and compared with baseline ECGs. The existence of ECG findings typical of severe hyperkalemia, or an alteration in ECG findings compared with previous results, was considered a 'change in ECG findings'. The findings considered typical of severe hyperkalemia were tall T waves, shortening of QT intervals, prolonged PR intervals, reductions in the amplitude of P waves, 'sine-wave' ventricular rhythms with wide QRS complexes, and the occurrences of ventricular fibrillation and asystole. Decisions on ECG findings were based on formal readings, documented by the attending cardiologist, and adjudicated by reviewers and researchers on the basis of an extensive literature review [14,22-24]. The period from the diagnosis of hyperkalemia to 'change in ECG findings' was also recorded.

Management of severe hyperkalemia was divided into a conservative management group and an aggressive management group; patients requiring RRT were in the aggressive management group. The choice of RRT modality was made by the attending physician after considering the clinical characteristics of each patient. The criteria for initiation of RRT in AKI included volume overload, oliguria, acidosis, refractory hyperkalemia, and uremic symptoms or documented uremia. We categorized management techniques into 'level of support I' and 'level of support II' categories. 'Level of support I' contained seven initial conservative management strategies, all given a weight of 1: drug cessation, intravenous (IV) calcium gluconate, dextrose fluid with insulin, IV or oral (PO) sodium bicarbonate, calcium polystyrene sulfonate enema, PO calcium polystyrene sulfonate, and IV or PO loop diuretics. The sum of weighted values was defined as the 'level of support I' value for each patient. 'Level of support II' contained nine initial conservative management strategies and RRT treatments: the previously named seven strategies were included with the addition of hemodialysis (weight 1) and continuous renal replacement therapy (CRRT) (weight 2). The sum of weighted values was defined as the 'level of support II' value for each patient.

Clinical parameters that could influence either the development of severe hyperkalemia or in-hospital mortality - serum creatinine, eGFR, total carbon dioxide, and arterial pH level - were documented. All laboratory data were collected from the time that the serum K^+ ^level reached at least 6.5 mEq/L. Serum creatinine levels were measured by using an assay based on the Jaffe method, and eGFR was calculated by using the following abbreviated Modification of Diet in Renal Disease formula: GFR (in mL/minute per 1.73 m^2^) = 186 × (serum creatinine) ^1.154 ^× (age in years)^-0.203 ^× (0.742 if female).

### Clinical outcomes

Clinical outcomes included ICU treatment (including reasons for ICU treatment), cardiopulmonary resuscitation (CPR), improvement in severe hyperkalemia, and in-hospital mortality (including reasons for in-hospital mortality). Improvement in hyperkalemia was defined as a serum K^+ ^level of less than 5.5 mEq/L, independent of deterioration in clinical condition or in-hospital mortality. In-hospital mortality was defined as death during the period of hospitalization, independent of an improvement in severe hyperkalemia.

### Statistical analysis

Categorical variables were described as the frequency and proportion of variables and were compared by using the chi-square test. Continuous variables were expressed as the mean ± standard deviation and compared by using a Student *t *test after normality testing. A simple logistic regression model was used to determine the unadjusted odds ratios (ORs) and 95% confidence intervals (CIs). A correlation analysis was conducted in order to avoid multi-collinearity; only one variable in highly correlated variable sets was selected for multiple logistic regression analysis. Statistically significant covariables from univariate analysis and clinically important covariables were included in the final multiple logistic regression model, conducted in a forward stepwise manner. The end results of multiple logistic regression analysis were demonstrated as a forest plot. A *P *value of less than 0.05 was considered statistically significant. Statistical analysis was performed with the Statistical Package for the Social Sciences, version 18.0K (SPSS, Inc., Chicago, IL, USA).

## Results

### Demographic and clinical baseline characteristics

**Figure 1:**
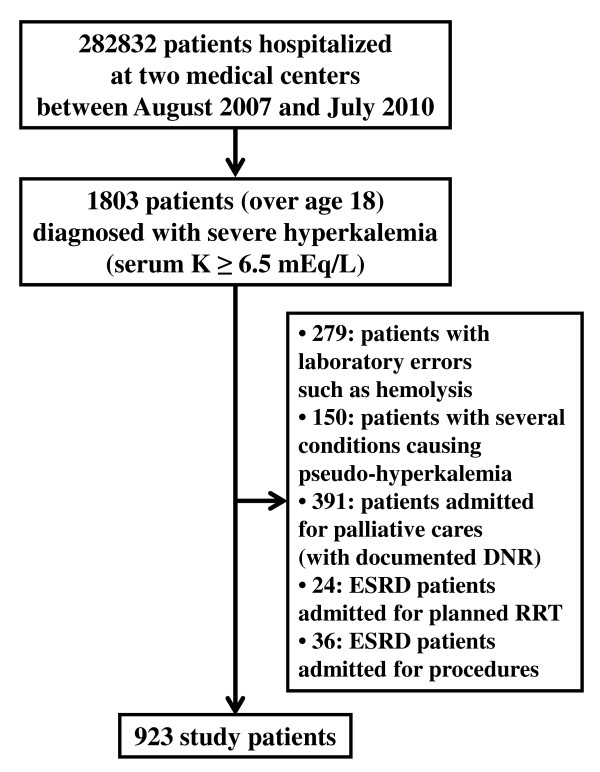
**Flow diagram for patient enrollment**. DNR, do not resuscitate; ESRD, end-stage renal disease; RRT, renal replacement therapy.

We identified 282,832 patients hospitalized at one of the two medical centers between August 2007 and July 2010 (Figure 1). Severe hyperkalemia was diagnosed in a total of 1,803 consecutive patients at least 19 years old. Patients were excluded from analysis if laboratory errors such as hemolysis were present (279 patients), if they had clinical conditions causing pseudohyperkalemia (150 patients), if they were admitted only for palliative care or had documented 'do not resuscitate' (DNR) status (391 patients), or if they were end-stage renal disease patients admitted for planned RRT (24 patients) or other procedures (36 patients). Thus, 923 patients were enrolled in this study.

The demographic and clinical baseline characteristics at the time of severe hyperkalemia diagnosis are shown in Table 1. Of the 923 patients, 586 (63.5%) were male and the mean age was approximately 61 years. The mean serum K^+ ^levels at admission and at the time of severe hyperkalemia diagnosis were 5.7 ± 1.5 mEq/L and 7.1 ± 0.7 mEq/L, respectively. Emergent admission was required in 68.6% of patients: 10.1% were admitted for severe hyperkalemia, and the remaining 89.9% were hospitalized for other causes, including non-severe hyperkalemia; the most common reason for admission was infection. Hyperkalemia occurred during the hospital course in 60.0% of patients; 40% of cases had an onset prior to admission. In those diagnosed during admission, the period from admission to diagnosis was approximately 17 days, and the most common hospital location at severe hyperkalemia diagnosis was the medical ward. Symptomatic patients accounted for 46.8% of those with severe hyperkalemia; the most common symptom was cardiac arrest, followed by arrhythmia and muscle weakness. MOF was present in 24.5% of patients at the time of hyperkalemia diagnosis, and 20.3% had cardiac arrest at the time of diagnosis.

**Table 1:** Demographic and clinical baseline characteristics

Characteristics	Number (percentage) unless indicated otherwise
Age, years^a^	61.1 ± 15.0

Male gender	586 (63.5)

Serum potassium level, mEq/L (K^+ ^≥6.5 mEq/L)^a^	7.1 ± 0.7

Serum potassium level at admission, mEq/L^a^	5.7 ± 1.5

The type of admission	

Planned admission	290 (31.4)

Emergent admission	633 (68.6)

Admission for severe hyperkalemia	93 (10.1)

Onset of hyperkalemia	

At the time of admission to the hospital	339 (40.0)

During the period of hospitalization	554 (60.0)

Period from admission to diagnosis, days^a^	16.7 ± 34.3

Location at diagnosis with hyperkalemia	

Intensive care unit	171 (30.9)

Surgical ward	111 (20.0)

Medical ward	244 (44.0)

Emergency room	28 (5.1)

Multi-organ failure at admission	108 (11.7)

Multi-organ failure at the time of diagnosis	226 (24.5)

Diagnosis at the time of cardiac arrest	187 (20.3)

Symptoms pertinent to hyperkalemia	432 (46.8)

Cardiac arrest	187 (43.3)

Arrhythmia	152 (35.2)

Other typical symptoms	93 (21.5)

Underlying diseases	

Diabetes mellitus	375 (40.6)

Hypertension	427 (46.3)

Chronic kidney disease (CKD)	648 (70.2)

Unknown stage	10 (1.5)

Stage II	158 (24.4)

Stage III	207 (31.9)

Stage IV	79 (12.3)

Stage V	194 (29.9)

ESRD on dialysis	160 (17.3)

Malignancy	299 (32.4)

Liver cirrhosis	161 (17.4)

Coronary artery disease	108 (11.7)

Pulmonary diseases	95 (10.3)

Cerebrovascular disease	95 (10.3)

History of recurrence for severe hyperkalemia	62 (6.7)

Congestive heart failure	71 (7.7)

Arrhythmia	

Atrial fibrillation	100 (10.8)

First degree atrioventricular block	27 (2.9)

Thyroid disease	38 (4.1)

Drugs	

Angiotensin-converting enzyme inhibitor	60 (6.5)

Angiotensin II receptor blocker	165 (17.9)

Potassium-sparing diuretics	108 (11.7)

Beta blocker	124 (13.4)

NSAIDs	22 (2.4)

Digoxin	25 (2.7)

Potassium supplements	129 (14.0)

Coexisting medical conditions	

1. Renal impairment	

New-onset acute kidney injury (AKI)	205 (22.2)

AKI superimposed on CKD	478 (51.8)

Infection	304 (32.9)

Volume depletion	426 (46.2)

Bleeding	173 (18.7)

2. Potassium shift from ICF to ECF	

Metabolic acidosis	592 (64.1)

Rhabdomyolysis	52 (5.6)

Tumor lysis syndrome	11 (1.2)

3. Others	

Poor compliance to K^+^-lowering agents	30 (3.3)

Constipation	7 (0.8)

Transfusion	24 (2.6)

Adrenal insufficiency	16 (1.7)

ECG changes pertinent to hyperkalemia	481/673 (71.5)

Period from diagnosis to ECG change, minutes^a^	21.6 ± 99.0

Typical findings	339 (70.5)

Atypical findings	142 (29.5)

^a^Mean ± standard deviation. ECF, extracellular fluid; ECG, electrocardiogram; ESRD, end-stage renal disease; ICF, intracellular fluid; NSAID, non-steroidal anti-inflammatory drug.

CKD was the most common underlying disease, and angiotensin II receptor blockers were the most common medications. AKI was a coexisting condition in 205 patients (22.2%) with normal baseline renal function and 478 patients (51.8%) with underlying CKD. In addition, 50.4% of all patients who underwent ECG demonstrated changes pertinent to severe hyperkalemia; the period from diagnosis with hyperkalemia to 'change in ECG findings' was approximately 22 minutes, and the most common findings were asystole and pulseless electrical activity.

### Severe hyperkalemia management and clinical outcomes

Calcium gluconate was used in 58.1% of patients, dextrose fluid mixed with insulin in 52.7%, and 38.4% received sodium bicarbonate (Table 2). Hyperkalemia-causing drugs were discontinued in 219 patients (23.7%). RRT was fairly common; 176 patients (19.1%) underwent hemodialysis and 71 (7.7%) received CRRT. The levels of support offered to patients are described in Additional file 1.

**Table 2:** Management and clinical outcomes of severe hyperkalemia

Management	Number (percentage) unless indicated otherwise
Conservative management	

Drug cessation	219 (23.7)

Calcium gluconate IV	536 (58.1)

Dextrose fluid + insulin	486 (52.7)

Sodium bicarbonate IV or PO	354 (38.4)

Calcium polystyrene sulfonate enema	279 (30.2)

Calcium polystyrene sulfonate PO	455 (49.3)

Loop diuretics IV or PO	98 (10.6)

Renal replacement therapy	

Hemodialysis	176 (19.1)

Continuous renal replacement therapy	71 (7.7)

Level of support I offered to patients^a^	2.6 ± 1.8

Level of support II offered to patients^a^	3.0 ± 1.9

	

Clinical outcomes	Number (percentage)

Intensive care unit (ICU) treatment	

No ICU care	601 (65.1)

Need for ICU care	126 (13.7)

During ICU care	196 (21.2)

Reasons for ICU admission	

Respiratory problem	146 (46.1)

Cardiac problem	26 (8.2)

Septic shock	32 (10.1)

Bleeding	17 (5.4)

Hemodynamic intensive monitoring	76 (24.0)

Others	20 (6.3)

Cardiopulmonary resuscitation (CPR)	

No CPR	631 (68.4)

CPR for issues related to severe hyperkalemia	60 (7.5)

CPR for other reasons	232 (25.1)

Improvement in severe hyperkalemia	715 (77.5)

In-hospital mortality (death)	283 (30.7)

Reasons for in-hospital mortality	

Respiratory problem	45 (15.9)

Cardiac problem	42 (14.8)

Septic shock	78 (27.6)

Progression of malignancy	20 (7.1)

Bleeding	35 (12.4)

Neurologic problem	20 (7.1)

Hepatic problem	23 (8.0)

Others	20 (7.1)

^a^Mean ± standard deviation. IV, intravenous; PO, by mouth (*per os*).

The lower part of Table 2 summarizes clinical outcomes during hospitalization. ICU treatment was required in 126 patients (13.7%), and the most common reason for ICU admission was respiratory compromise, followed by the need for hemodynamic intensive monitoring and septic shock. CPR was administered for issues related to severe hyperkalemia in 60 patients (6.5%); 232 patients (25.1%) had CPR for other reasons. Severe hyperkalemia improved in 715 patients (77.5%), and a total of 283 patients (30.7%) died. The most common reason for in-hospital mortality was septic shock, followed by respiratory and cardiac issues.

As shown in Additional file 2, patients diagnosed at the time of cardiac arrest and those with MOF at the time of diagnosis had lower improvement rates than those who did not have these complications at the time of diagnosis. We also analyzed the association of the level of support offered to patients and their clinical outcomes, including improvement in hyperkalemia and in-hospital mortality (Table 3). Higher values for the level of support, in both categories I and II, were significantly associated with improvement in hyperkalemia. The value for level of support I was higher in the survival group than the in-hospital mortality group; level of support II value was not associated with in-hospital mortality. In other words, aggressive initial treatments resulted in improvement of hyperkalemia and higher survival rate. In patients receiving even RRT with initial treatments, hyperkalemia improved; however, in-hospital mortality was not affected.

**Table 3:** Association of level of support offered to patients and clinical outcomes

	Improvement in hyperkalemia			In-hospital mortality		
	**Improvement** **(*n *= 715)**	**No improvement** **(*n *= 208)**	***P***	**Death** **(*n *= 283)**	**Survival** **(*n *= 640)**	***P***

Level of support I^a^	2.8 ± 1.8	2.2 ± 1.5	< 0.001	2.4 ± 1.5	2.7 ± 1.8	0.012

Level of support II^b^	3.1 ± 1.9	2.6 ± 1.8	< 0.001	2.9 ± 1.9	3.0 ± 1.9	0.266

Values are presented as mean ± standard deviation. ^a^Defined as the sum of weighted value for initial conservative management offered to each patient, including drug cessation (weight 1), intravenous (IV) calcium gluconate (weight 1), dextrose fluid with insulin (weight 1), IV or oral sodium bicarbonate (weight 1), calcium polystyrene sulfonate enema (weight 1), oral calcium polystyrene sulfonate (weight 1), and IV or oral loop diuretics (weight 1). ^b^Defined as the sum of weighted value for initial conservative management and renal replacement therapy offered to each patient, including hemodialysis (weight 1) and continuous renal replacement therapy (weight 2).

### Association between in-hospital mortality and clinical factors

Tables 4 and 5 show the comparison between the survival group and the in-hospital mortality group, and the association of clinical factors, both modifiable and non-modifiable, with in-hospital mortality. The OR for mortality increased as the levels of the following modifiable factors increased: the serum K^+ ^level and the difference between serum K^+ ^levels at admission and at its highest point. Severe medical conditions, including infection, volume depletion, and bleeding, were significantly associated with a higher mortality rate. Furthermore, the development of AKI in patients with normal baseline renal function was a clear predictor of a higher mortality rate (OR 5.23, 95% CI 3.75 to 7.30; *P *< 0.001). In contrast, the mortality rate decreased in patients with AKI superimposed on CKD (OR 0.53, 95% CI 0.40 to 0.70; *P *< 0.001). These findings are demonstrated in Table 6, which evaluates the mortality rate in patients with AKI according to the presence or absence of underlying CKD. Patients with AKI superimposed on CKD had much lower mortality rates than those with AKI developing from normal baseline renal function (OR 0.42, 95% CI 0.23 to 0.74; *P *= 0.003) (Table 6). Patients who received CPR had much higher mortality rates than those who did not; in particular, CPR significantly increased the mortality rate when performed for causes other than those related to hyperkalemia. ICU treatment during hospitalization was also significantly associated with higher in-hospital mortality.

**Table 4:** Association between in-hospital mortality and clinical factors

	Number (percentage) unless indicated otherwise		Univariate analysis		Multiple logistic regression analysis^a^	
	**In-hospital mortality group**	**Survival group**	**OR (95% CI)**	***P***	**OR (95% CI)**	***P***

Modifiable factors						

Serum K level, mEq/L^b^	7.3 ± 0.9	7.0 ± 0.6	1.66 (1.37-2.00)	< 0.001		

△Serum K level, mEq/L^b^	2.2 ± 1.5	1.1 ± 1.3	1.70 (1.53-1.89)	< 0.001	1.83 (1.52-2.20)	< 0.001

Coexisting medical conditions^c^						

New-onset AKI	120 (42.4)	79 (12.3)	5.23 (3.75-7.30)	< 0.001	2.17 (1.27-3.71)	0.005

AKI on CKD	117 (41.3)	366 (57.2)	0.53 (0.40-0.70)	< 0.001		

Infection	155 (54.8)	149 (23.3)	3.99 (2.96-5.37)	< 0.001	2.07 (1.27-3.38)	0.004

Volume depletion	165 (58.3)	261 (40.8)	2.03 (1.53-2.70)	< 0.001		

Bleeding	108 (38.2)	65 (10.2)	5.46 (3.84-7.76)	< 0.001	4.56 (2.61-7.98)	< 0.001

Rhabdomyolysis	39 (13.8)	13 (2.0)	7.71 (4.05-14.69)	< 0.001		

Tumor lysis syndrome	9 (3.2)	2 (0.3)	10.48 (2.25-48.81)	0.003		

Poor compliance	4 (1.4)	26 (4.1)	0.34 (0.12-0.98)	0.046		

Constipation	2 (0.7)	5 (0.8)	0.90 (0.17-4.69)	0.904		

Transfusion	12 (4.2)	12 (1.9)	2.32 (1.03-5.22)	0.043		

Adrenal insufficiency	4 (1.4)	12 (1.9)	0.75 (0.24-2.35)	0.621		

CPR						

No indication	22 (7.8)	609 (95.2)	Reference			

Due to hyperkalemia	47 (16.6)	13 (2.0)	100.1 (47.4-211.3)	< 0.001		

Due to other causes	214 (75.6)	18 (2.8)	329.1 (173.2- 625.5)	< 0.001		

ICU treatment						

No indication	83 (29.3)	518 (80.9)	Reference		Reference	

Need for ICU care	80 (28.3)	46 (7.2)	10.85 (7.06-16.69)	< 0.001	3.62 (1.79-7.32)	< 0.001

During ICU care	120 (42.4)	76 (11.9)	9.85 (6.81-14.25)	< 0.001	2.98 (1.69-5.24)	< 0.001

Level of support I^b^	2.4 ± 1.5	2.7 ± 1.8	0.91 (0.84-0.99)	0.020		

Level of support II^b^	2.9 ± 1.9	3.0 ± 1.9	0.96 (0.89-1.03)	0.266		

Improvement in hyperkalemia^c^	132 (46.6)	583 (91.1)	0.09 (0.06-0.12)	< 0.001		

						

Non-modifiable factors						

Male gender	199 (70.3)	387 (60.5)	1.55 (1.15-2.09)	0.004		

Age, years^b^	60.6 ± 15.5	61.3 ± 14.7	1.00 (0.99 - 1.01)	0.502		

Underlying diseases^c^						

Diabetes mellitus	82 (29.0)	293 (45.8)	0.48 (0.36-0.65)	< 0.001		

Hypertension	90 (31.8)	337 (52.7)	0.42 (0.31-0.56)	< 0.001		

Chronic kidney disease (CKD)						

No CKD + stage I	139 (49.3)	136 (21.6)	Reference			

Stage II	55 (19.5)	103 (16.3)	0.52 (0.35-0.78)	0.002		

Stage III	50 (17.7)	157 (24.9)	0.31 (0.21-0.46)	< 0.001		

Stage IV	9 (3.2)	70 (11.1)	0.13 (0.06-0.26)	< 0.001		

Stage V	29 (10.3)	165 (26.1)	0.17 (0.11-0.27)	< 0.001		

Malignancy	114 (40.3)	185 (28.9)	1.66 (1.24-2.22)	0.001	2.88 (1.68-4.96)	< 0.001

Liver cirrhosis	55 (19.4)	106 (16.6)	1.21 (0.85-1.74)	0.290		

CHF	24 (8.5)	47 (7.3)	1.16 (0.69-1.94)	0.583		

Arrhythmia						

Atrial fibrillation	37 (13.1)	63 (9.8)	1.39 (0.90-2.14)	0.141		

SSS, 1' AV block	9 (3.2)	18 (2.8)	1.18 (0.52-2.66)	0.691		

Thyroid disease	11 (3.9)	27 (4.2)	0.92 (0.45-1.87)	0.815		

Coronary artery disease	31 (11.0)	77 (12.0)	0.91 (0.58-1.42)	0.682		

Pulmonary disease	33 (11.7)	62 (9.7)	1.23 (0.79-1.93)	0.364		

Cerebrovascular disease	26 (9.2)	69 (10.8)	0.84 (0.52-1.35)	0.463		

History of recur	6 (2.1)	56 (8.8)	0.23 (0.10-0.53)	0.001		

The type of admission						

Planned admission	38 (13.4)	252 (39.4)	Reference		Reference	

Emergent admission	245 (86.6)	388 (60.6)	4.19 (2.87-6.10)	< 0.001	2.97 (1.56-5.66)	0.001

Onset of hyperkalemia						

On admission	69 (24.4)	300 (46.9)	Reference			

During admission	214 (75.6)	340 (53.1)	2.74 (2.00-3.74)	< 0.001		

Location at diagnosis with hyperkalemia						

Emergency room	68 (24.0)	182 (28.4)	Reference			

ICU	118 (41.7)	74 (11.6)	4.27 (2.85-6.38)	< 0.001		

Surgical ward	11 (3.9)	138 (21.6)	0.21 (0.11-0.42)	< 0.001		

Medical ward	86 (30.4)	246 (38.4)	0.94 (0.65-1.36)	0.726		

MOF at admission^c^	71 (25.1)	37 (5.8)	5.46 (3.56-8.37)	< 0.001		

MOF at diagnosis^c^	194 (68.6)	32 (5.0)	41.42 (26.80-63.99)	< 0.001	7.64 (4.00-14.57)	< 0.001

Diagnosis at arrest^c^	166 (58.7)	21 (3.3)	41.82 (25.49-68.61)	< 0.001		

Symptoms pertinent to hyperkalemia						

Asymptomatic	77 (27.2)	414 (64.5)	Reference		Reference	

Cardiac arrest	166 (58.7)	21 (3.4)	42.96 (25.65-71.94)	< 0.001	8.84 (4.18-18.68)	< 0.001

Arrhythmia	30 (10.6)	122 (19.1)	1.50 (0.94-2.40)	0.087	1.24 (0.63-2.43)	0.533

Other symptoms	10 (3.5)	83 (13.0)	0.58 (0.29-1.16)	0.123	0.64 (0.26-1.59)	0.338

^a^Covariables: gender, age, serum K^+ ^level, the differences between the admission and highest serum K^+ ^levels, diabetes mellitus, hypertension, malignancy, history of recurrence, angiotensin-converting enzyme inhibitor, angiotensin II receptor blocker, beta blocker, K^+^-sparing diuretics, non-steroidal anti-inflammatory drugs, infection, volume depletion, bleeding, poor compliance, transfusion, new-onset acute kidney injury (AKI), AKI on chronic kidney disease, multi-organ failure (MOF) at the time of diagnosis, the type of admission, onset of hyperkalemia, symptoms pertinent to hyperkalemia, level of support I, and intensive care unit (ICU) treatment. ^b^Mean ± standard deviation. ^c^The following were entered as 'yes-no' variables: underlying diseases; coexisting medical conditions; drug-induced hyperkalemia; potassium supplements; the presence of multiple organ failure at admission or at hyperkalemia diagnosis; hyperkalemia diagnosis at the time of cardiac arrest; and improvement in hyperkalemia. The frequency, proportion, and odds ratios (ORs) of these variables were determined by comparing 'yes' variables to the 'no' variables. AV, atrioventricular; CHF, congestive heart failure; CI, confidence interval; CPR, cardiopulmonary resuscitation; SSS, sick sinus syndrome.

**Table 5:** Association of in-hospital mortality and drugs or electrocardiogram findings at hyperkalemia diagnosis

	Number (percentage)		Univariate analysis		Multiple logistic regression analysis^a^	
	**In-hospital mortality group**	**Survival group**	**OR (95% CI)**	***P***	**OR (95% CI)**	***P***

Modifiable factors						

Drug-induced hyperkalemia^b^						

ACEi	7 (2.5)	53 (8.3)	0.28 (0.13-0.63)	0.002		

ARB	19 (6.7)	146 (22.8)	0.24 (0.15-0.40)	< 0.001		

Beta blocker	17 (6.0)	107 (16.7)	0.32 (0.19-0.54)	< 0.001	0.31 (0.13-0.74)	0.009

K^+^-sparing diuretics	20 (7.1)	88 (13.8)	0.48 (0.29-0.79)	0.004		

NSAIDs	2 (0.7)	20 (3.1)	0.22 (0.05-0.95)	0.043		

Digoxin	9 (3.2)	16 (2.5)	1.28 (0.56-2.94)	0.558		

K supplements^b^	38 (13.4)	91 (14.2)	0.94 (0.62-1.41)	0.749		

				

Non-modifiable factors						

ECG findings pertinent to hyperkalemia						

No changes	23 (8.7)	169 (41.3)	Reference			

Atypical findings	45 (17.0)	97 (23.7)	3.37 (1.93-5.88)	< 0.001		

Typical findings	196 (74.2)	143 (35.0)	9.36 (5.80-15.10)	< 0.001		

^a^Covariables: gender, age, serum K^+ ^level, the differences between the admission and highest serum K^+ ^levels, diabetes mellitus, hypertension, malignancy, history of recurrence, angiotensin-converting enzyme inhibitor (ACEi), angiotensin II receptor blocker (ARB), beta blocker, K^+^-sparing diuretics, non-steroidal anti-inflammatory drugs (NSAIDs), infection, volume depletion, bleeding, poor compliance, transfusion, new-onset acute kidney injury, acute kidney injury on chronic kidney disease, multi-organ failure at the time of diagnosis, the type of admission, onset of hyperkalemia, symptoms pertinent to hyperkalemia, level of support I, and intensive care unit treatment. ^b^The following were entered as 'yes-no' variables: underlying diseases; coexisting medical conditions; drug-induced hyperkalemia; potassium supplements; the presence of multiple organ failure at admission or at hyperkalemia diagnosis; hyperkalemia diagnosis at the time of cardiac arrest; and improvement in hyperkalemia. The frequency, proportion, and odds ratios (ORs) of these variables were determined by comparing 'yes' variables to the 'no' variables. CI, confidence interval; ECG, electrocardiogram.

**Table 6:** Association of in-hospital mortality and acute kidney injury and underlying chronic kidney disease

		Univariate analysis		Multiple logistic regression analysis^a^	
	**Number**	**OR (95% CI)**	***P***	**OR (95% CI)**	***P***

CKD^- ^AKI^+^	199	Reference		Reference	

CKD^- ^AKI^-^	76	0.22 (0.12-0.40)	< 0.001	0.65 (0.26-1.62)	0.357

CKD^+ ^AKI^-^	165	0.13 (0.08-0.21)	< 0.001	0.52 (0.25-1.12)	0.095

CKD^+ ^AKI^+^	483	0.21 (0.15-0.30)	< 0.001	0.42 (0.23-0.74)	0.003

^a^Covariables: gender, age, serum K^+ ^level, the differences between the admission and highest serum K^+ ^levels, diabetes mellitus, hypertension, malignancy, history of recurrence, angiotensin-converting enzyme inhibitor, angiotensin II receptor blocker, beta blocker, K^+^-sparing diuretics, non-steroidal anti-inflammatory drugs, infection, volume depletion, bleeding, poor compliance, transfusion, new-onset acute kidney injury (AKI), AKI on chronic kidney disease (CKD), multi-organ failure at the time of diagnosis, the type of admission, onset of hyperkalemia, symptoms pertinent to hyperkalemia, level of support I, and intensive care unit treatment. CI, confidence interval; OR, odds ratio.

With respect to drug-induced hyperkalemia, the in-hospital mortality rate was lower for those receiving angiotensin-converting enzyme inhibitors, angiotensin II receptor blockers, beta blockers, K^+^-sparing diuretics, and non-steroidal anti-inflammatory drugs (Table 5). Moreover, a higher value for level of support I, indicating aggressive initial treatment, was associated with a lower mortality rate (OR 0.91, 95% CI 0.84 to 0.99; *P *= 0.020). Improvement in hyperkalemia also markedly lowered in-hospital mortality (OR 0.09, 95% CI 0.06 to 0.12; *P *< 0.001).

Of the non-modifiable factors, male gender and underlying malignancy were significant risk factors for higher in-hospital mortality. The mortality rate was lower, however, in patients with diabetes, hypertension, and a history of recurrent severe hyperkalemia. As CKD progressed to higher stages, the OR of in-hospital mortality decreased: stage II, OR 0.52, 95% CI 0.35 to 0.78; *P *= 0.002; stage III, OR 0.31, 95% CI 0.21 to 0.46; *P *< 0.001; stage IV, OR 0.13, 95% CI 0.06 to 0.26; *P *< 0.001; stage V, OR 0.17, 95% CI 0.11 to 0.27; *P *< 0.001. Age and underlying congestive heart failure did not influence in-hospital mortality.

The in-hospital mortality rate was strongly correlated with ECG changes pertinent to hyperkalemia, MOF at admission, and emergent admission (OR 4.29, 95% CI 2.87 to 6.10; *P *< 0.001). The factors of hospital-acquired hyperkalemia, presence of MOF at the time of diagnosis, diagnosis at the time of cardiac arrest, and cardiac arrest as a symptom of hyperkalemia were also significantly associated with higher in-hospital mortality.

**Figure 2:**
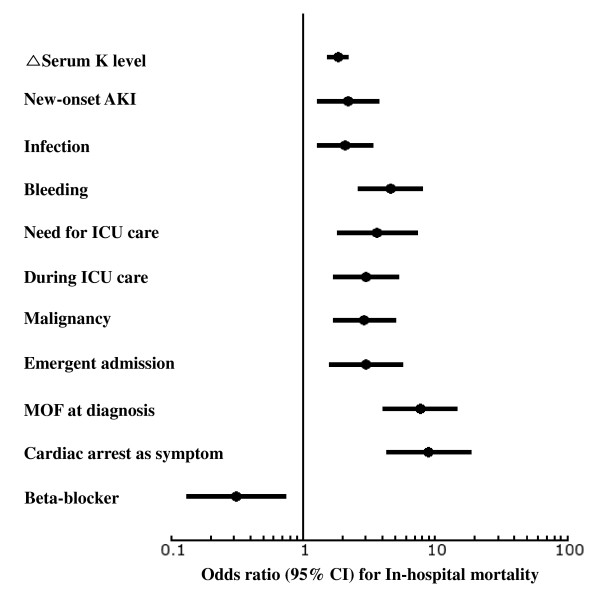
**Risk factors for in-hospital mortality**. Malignancy, emergent admission, the presence of multi-organ failure (MOF) and/or cardiac arrest at the time of hyperkalemia diagnosis, intensive care unit (ICU) care during hospitalization, and severe clinical situations such as new-onset acute kidney injury (AKI), infection, and bleeding were strongly associated with in-hospital mortality. The mortality rate increased significantly as the difference in serum K^+ ^level at admission and at its highest point (serum K^+ ^≥ 6.5 mEq/L) increased. In contrast, the mortality rate decreased in patients with drug-induced hyperkalemia, including those who used beta blockers. CI, confidence interval.

Multiple logistic regression analysis demonstrated that malignancy and the precipitating conditions of new-onset AKI, infection, and bleeding were strong risk factors for in-hospital mortality. Furthermore, it was verified that in-hospital mortality increased significantly as the difference between serum K^+ ^levels at admission and its highest levels increased. Emergent admission, the presence of MOF at the time of diagnosis, cardiac arrest as a symptom of hyperkalemia, and ICU care during hospitalization were also associated with a higher mortality rate. In contrast, the mortality rate was lower in patients with drug-induced hyperkalemia (Figure 2).

## Discussion

This study was designed to identify the principal factors predisposing to severe hyperkalemia and to analyze the relationship between the serum K^+ ^level and clinical outcomes, including in-hospital mortality. We also investigated the association between in-hospital mortality and multiple clinical factors in patients with severe hyperkalemia.

Severe hyperkalemia was shown to result from various medical conditions, predisposing factors, and medications. Despite appropriate and aggressive management, the in-hospital mortality was very high (30.7%). The meaningful risk factors for in-hospital mortality were greater differences in serum K^+ ^levels at admission and its highest levels, underlying malignancy, precipitating factors such as infection and bleeding, emergent admission, the presence of MOF at hyperkalemia diagnosis, the occurrence of cardiac arrest as a symptom of hyperkalemia, and ICU treatment. Also notable is the fact that patients with normal baseline renal function had a much higher mortality rate. In contrast, the mortality rate decreased in patients with drug-induced hyperkalemia. Aggressive initial treatment for severe hyperkalemia resulted in improvement of K^+ ^values and consequently lowered the in-hospital mortality rate.

The results of the present study correspond well with those of earlier studies which reported that angiotensin-converting enzyme inhibitors, K^+^-sparing diuretics, non-steroidal anti-inflammatory drugs, and K^+ ^supplements are associated with an increased incidence of hyperkalemia [25,26]. In addition, our results agree with several published studies that factors such as diabetes, congestive heart failure, liver cirrhosis, and metabolic acidosis may contribute to the development of hyperkalemia [27-30]. The association between renal impairment and hyperkalemia is well documented [31-33]. Our findings are consistent with those of prior studies indicating that the mortality rate in patients with hyperkalemia increases as the serum K^+ ^level increases [34,35]. Additionally, the finding that most cases resulting in death are complicated by other medical conditions such as renal failure and metabolic acidosis is supported by the present study [34]. The results of our study coincide well with those of Evans and colleagues [36], showing that ECG changes that are more severe, such as cardiac arrest, are associated with greater degrees of hyperkalemia and with a higher mortality rate.

The present study has a different level of significance compared with other studies, as we demonstrated a negative association between baseline renal function and in-hospital mortality. Whereas the development of AKI superimposed on CKD was associated with a better prognosis, the occurrence of AKI in patients with normal baseline renal function conferred an increased mortality rate and a poorer prognosis. Similarly, in the group of patients without AKI, the in-hospital mortality rate was higher in patients without underlying CKD. Patients with CKD are exposed to hyperkalemia over a long period of time, and thus the normal range of serum K^+ ^levels in such patients should be considered higher than in other patients; an ability to adapt to an imbalance in the serum K^+ ^level may be acquired, making the clinical manifestations of severe hyperkalemia, and its outcomes, relatively mild and less harmful.

In the same vein, a large number of patients with underlying diabetes and hypertension had renal insufficiency, and their mortality rate during the period of hospitalization tended to be lower than that of other patients. Similarly, patients taking offending drugs had baseline renal impairment, and thus the mortality rate appeared to decrease. Indeed, the mortality rate was much higher in patients with AKI developing from normal baseline renal function, because of a lack of ability of these patients to adapt to higher serum K^+ ^levels.

We also demonstrated that the severe medical conditions of infection and bleeding, accompanying hyperkalemia, increased the mortality rate; these findings had clear significance after adjustment for serum K^+ ^levels. The typical ECG changes and symptoms pertinent to hyperkalemia, particularly cardiac arrest, were shown to be associated with a higher mortality rate as well. Death of patients with severe hyperkalemia was not attributable solely to the severity of the hyperkalemia but to the severity of the coexisting medical conditions. Supporting these findings, the presence of MOF at hyperkalemia diagnosis and the need for emergent admission or ICU treatment (or for both) were strongly correlated with an increased mortality rate. These factors were independently associated with the severity of the accompanying clinical situation rather than with the severe hyperkalemia itself.

There are several limitations in our study. First, the entire study population was hospitalized and diagnosed with severe hyperkalemia; there was no control group. It is therefore impossible to compare various characteristics between our patients and those with normal K^+ ^levels. Second, there is a potentially inherent bias resulting from the fact that only attending physicians evaluated the patients and classified the diverse clinical situations. Although researchers examined and reviewed the data from the electronic medical record in an attempt to minimize bias, inevitable limitations exist. Third, this study was based upon the premise that all patients, except those with DNR status, received equally effective treatment for other conditions such as infection, volume depletion, and bleeding; we did not verify all treatments offered to all patients. Hence, the association between treatments not related to hyperkalemia and clinical outcomes was not verified. Fourth, it was difficult to identify accurately the drugs that caused severe hyperkalemia, as most patients took a number of prescribed medications concurrently. Fifth, the ECG changes described could also have resulted from severe acidosis and myocardial ischemia. For this reason, it was difficult to estimate whether ECG changes were due to severe hyperkalemia alone. Finally, the present study was retrospective in nature, using only an electronic medical record system, and thus some essential information was often unavailable and the subjective opinions and decisions of the researcher became involved in data collection and analysis. Our retrospective study also may have inherent confounders for mortality, compromising our ability to identify a causal relationship between various clinical factors and the in-hospital mortality rate. Accordingly, well-designed, multicenter, prospective, large cohort studies should be conducted to verify the implications of numerous predisposing factors involving medications, comorbidities, and concurrent medical conditions. These future studies should also evaluate the role of different treatments in reducing mortality.

## Conclusions

Severe hyperkalemia, requiring hospitalization and prompt treatment, occurs in patients with diverse medical conditions; precipitating factors also vary. The mortality rate increases in patients with greater differences between the admission and highest serum K^+ ^levels, in patients with MOF or cardiac arrest (or both) at the time of hyperkalemia diagnosis, in those with severe underlying diseases, in those with coexisting medical conditions, and in those who develop AKI from normal baseline renal function, as opposed to those with underlying CKD, with or without AKI.

Consequently, controlling and maintaining the serum K^+ ^level within a safe range have great importance in the clinical setting. Determining additional risk factors for severe hyperkalemia may be valuable and instructive to clinicians in the identification of high-risk patients and in the effective management of severe hyperkalemia.

## Key messages

• Severe hyperkalemia occurs with diverse precipitating factors in patients with various medical conditions.

• An increased in-hospital mortality rate is significantly associated with severe underlying disease and coexisting medical conditions as well as with severe hyperkalemia itself.

• More importantly, the mortality rate is higher in patients with normal baseline renal function than in those with underlying CKD.

## Abbreviations

AKI: acute kidney injury; CI: confidence interval; CKD: chronic kidney disease; CPR: cardiopulmonary resuscitation; CRRT: continuous renal replacement therapy; DNR: do not resuscitate; ECG: electrocardiogram; eGFR: estimated glomerular filtration rate; ICU: intensive care unit; IV: intravenous; MOF: multi-organ failure; OR: odds ratio; PO: by mouth (*per os*); RRT: renal replacement therapy.

## Competing interests

The authors declare that they have no competing interests.

## Authors' contributions

JA participated in the design of the study, reviewed and collected data by using an electronic medical records system, performed the statistical analysis, and drafted the manuscript. JL carried out analysis and interpretation of data, helped to draft the manuscript, and revised it. HJ participated in acquisition of data and statistical analysis. DK participated in the design of the study, analysis, and interpretation of data. YO participated in the design of the study and acquisition of data. YK participated in the conception of the study, acquisition of data, and helped to draft the manuscript. CL had made substantial contributions to the conception and design of the study and to the drafting and revising of the manuscript. All authors read and approved the final manuscript.

## Supplementary Material

Additional file 1 **Level of Support Offered to Patients Diagnosed with Severe Hyperklaemia**. We categorized management techniques into 'level of support I' and 'level of support II' categories. 'Level of support I' contained 7 initial conservative management strategies, all given a weight of 1: drug cessation; intravenous (IV) calcium gluconate; dextrose fluid with insulin; IV or oral (PO) sodium bicarbonate; calcium polystyrene sulfonate enema; PO calcium polystyrene sulfonate; and IV or PO loop diuretics. The sum of weighted values was defined as the 'level of support I' value for each patient. 'Level of support II' contained 9 initial conservative management strategies and RRT treatments: the previously named 7 strategies were included, with the addition of hemodialysis (weight 1); and continuous renal replacement therapy (CRRT) (weight 2). The sum of weighted values was defined as the 'level of support II' value for each patient. The levels of support offered to patients are described in Additional file 1. Click here for file

Additional file 2 **Association between improvement in severe hyperkalemia and deterioration occurring at the time of diagnosis**. Patients diagnosed at the time of cardiac arrest, and those with MOF at the time of diagnosis, had lower improvement rates than those who did not have these complications at the time of diagnosis. Furthermore, improvement in hyperkalemia also markedly lowered in-hospital mortality. Click here for file

## References

[B1] Giebisch G Renal potassium transport: mechanisms and regulation Am J Physiol 1998 16 F817 F833 9612319 10.1152/ajprenal.1998.274.5.F817

[B2] Macdonald JE Struthers AD What is the optimal serum potassium level in cardiovascular patients? J Am Coll Cardiol 2004 16 155 161 10.1016/j.jacc.2003.06.021 14736430

[B3] Hoskote SS Joshi SR Ghosh AK Disorders of potassium homeostasis: pathophysiology and management J Assoc Physicians India 2008 16 685 693 19086355

[B4] Perazella MA Mahnensmith RL Hyperkalemia in the elderly: drugs exacerbate impaired potassium homeostasis J Gen Intern Med 1997 16 646 656 10.1046/j.1525-1497.1997.07128.x 9346463 PMC1497179

[B5] Tran HA Extreme hyperkalemia South Med J 2005 16 729 732 10.1097/01.SMJ.0000149407.51134.77 16108244

[B6] Reardon LC Macpherson DS Hyperkalemia in outpatients using angiotensin-converting enzyme inhibitors. How much should we worry? Arch Intern Med 1998 16 26 32 10.1001/archinte.158.1.26 9437375

[B7] Rimmer JM Horn JF Gennari FJ Hyperkalemia as a complication of drug therapy Arch Intern Med 1987 16 867 869 10.1001/archinte.1987.00370050063011 3579440

[B8] Borra S Shaker R Kleinfeld M Hyperkalemia in an adult hospitalized population Mt Sinai J Med 1988 16 226 229 3260990

[B9] Acker CG Johnson JP Palevsky PM Greenberg A Hyperkalemia in hospitalized patients: causes, adequacy of treatment, and results of an attempt to improve physician compliance with published therapy guidelines Arch Intern Med 1998 16 917 924 10.1001/archinte.158.8.917 9570179

[B10] Mahoney BA Smith WA Lo DS Tsoi K Tonelli M Clase CM Emergency interventions for hyperkalaemia Cochrane Database Syst Rev 2005 CD003235 15846652 10.1002/14651858.CD003235.pub2 PMC6457842

[B11] Shemer J Modan M Ezra D Cabili S Incidence of hyperkalemia in hospitalized patients Isr J Med Sci 1983 16 659 661 6885354

[B12] Ohmae M Rabkin SW Hyperkalemia-induced bundle branch block and complete heart block Clin Cardiol 1981 16 43 46 10.1002/clc.4960040110 7226590

[B13] Spodick DH Effects of severe hyperkalemia Am Heart Hosp J 2008 16 68 10.1111/j.1751-7168.2008.07777.x 18256562

[B14] Mattu A Brady WJ Robinson DA Electrocardiographic manifestations of hyperkalemia Am J Emerg Med 2000 16 721 729 10.1053/ajem.2000.7344 11043630

[B15] Ettinger PO Regan TJ Oldewurtel HA Hyperkalemia, cardiac conduction, and the electrocardiogram: a review Am Heart J 1974 16 360 371 10.1016/0002-8703(74)90473-6 4604546

[B16] Gennari FJ Disorders of potassium homeostasis. Hypokalemia and hyperkalemia Crit Care Clin 2002 16 273 288 10.1016/S0749-0704(01)00009-4 12053834

[B17] Jackson MA Lodwick R Hutchinson SG Hyperkalaemic cardiac arrest successfully treated with peritoneal dialysis Br Med J 1996 16 1289 1290 10.1136/bmj.312.7041.1289 8634622 PMC2351090

[B18] Voelckel W Kroesen G Unexpected return of cardiac action after termination of cardiopulmonary resuscitation Resuscitation 1996 16 27 29 10.1016/0300-9572(96)00954-9 8809916

[B19] Niemann JT Cairns CB Hyperkalemia and ionized hypocalcemia during cardiac arrest and resuscitation: possible culprits for postcountershock arrhythmias? Ann Emerg Med 1999 16 1 7 10.1016/S0196-0644(99)70265-9 10381988

[B20] Bellomo R Ronco C Kellum JA Mehta RL Palevsky P Acute renal failure - definition, outcome measures, animal models, fluid therapy and information technology needs: the Second International Consensus Conference of the Acute Dialysis Quality Initiative (ADQI) Group Crit Care 2004 16 R204 R212 10.1186/cc2872 15312219 PMC522841

[B21] Mehta RL Kellum JA Shah SV Molitoris BA Ronco C Warnock DG Levin A Acute Kidney Injury Network: report of an initiative to improve outcomes in acute kidney injury Crit Care 2007 16 R31 10.1186/cc5713 17331245 PMC2206446

[B22] Diercks DB Shumaik GM Harrigan RA Brady WJ Chan TC Electrocardiographic manifestations: electrolyte abnormalities J Emerg Med 2004 16 153 160 10.1016/j.jemermed.2004.04.006 15261358

[B23] Webster A Brady W Morris F Recognising signs of danger: ECG changes resulting from an abnormal serum potassium concentration Emerg Med J 2002 16 74 77 10.1136/emj.19.1.74 11777886 PMC1725789

[B24] Yu AS Atypical electrocardiographic changes in severe hyperkalemia Am J Cardiol 1996 16 906 908 10.1016/S0002-9149(97)89197-7 8623755

[B25] Beroniade V Cornielle L Haraoui B Indomethacin-induced inhibition of prostaglandin with hyperkalemia Ann Intern Med 1979 16 499 500 475198 10.7326/0003-4819-91-3-499_2

[B26] DuBose TD Jr Hyperkalemic hyperchloremic metabolic acidosis: pathophysiologic insights Kidney Int 1997 16 591 602 10.1038/ki.1997.85 9027745

[B27] Wrenger E Müller R Moesenthin M Welte T Frölich JC Neumann KH Interaction of spironolactone with ACE inhibitors or angiotensin receptor blockers: analysis of 44 cases Br Med J 2003 16 147 149 10.1136/bmj.327.7407.147 12869459 PMC1126510

[B28] Ramadan FH Masoodi N El-Solh AA Clinical factors associated with hyperkalemia in patients with congestive heart failure J Clin Pharm Ther 2005 16 233 239 10.1111/j.1365-2710.2005.00638.x 15896240

[B29] Henz S Maeder MT Huber S Schmid M Loher M Fehr T Influence of drugs and comorbidity on serum potassium in 15 000 consecutive hospital admissions Nephrol Dial Transplant 2008 16 3939 3945 10.1093/ndt/gfn380 18614817

[B30] Sato A Saruta T Aldosterone-induced organ damage: plasma aldosterone level and inappropriate salt status Hypertens Res 2004 16 303 310 10.1291/hypres.27.303 15198476

[B31] Stevens MS Dunlay RW Hyperkalemia in hospitalized patients Int Urol Nephrol 2000 16 177 180 10.1023/A:1007135517950 11229629

[B32] Hu Y Carpenter JP Cheung AT Life-threatening hyperkalemia: a complication of spironolactone for heart failure in a patient with renal insufficiency Anesth Analg 2002 16 39 41 10.1097/00000539-200207000-00006 12088939

[B33] Cruz CS Cruz AA Marcilio de Souza CA Hyperkalaemia in congestive heart failure patients using ACE inhibitors and spironolactone Nephrol Dial Transplant 2003 16 1814 1819 10.1093/ndt/gfg295 12937229

[B34] Paice B Gray JM McBride C Donnelly T Lawson DH Hyperkalemia in patients in hospital Br Med J 1983 16 1189 1192 10.1136/bmj.286.6372.1189 6404388 PMC1547392

[B35] Takaichi K Takemoto F Ubara Y Mori Y Analysis of factors causing hyperkalemia Intern Med 2007 16 823 829 10.2169/internalmedicine.46.6415 17575373

[B36] Evans K Reddan DN Szczech LA Nondialytic management of hyperkalemia and pulmonary edema among end-stage renal disease patients: an evaluation of the evidence Semin Dial 2004 16 22 29 10.1111/j.1525-139X.2004.17110.x 14717808

